# Application of Fiber Bragg Gratings as a Sensor of Pulsed Mechanical Action

**DOI:** 10.3390/s22197289

**Published:** 2022-09-26

**Authors:** Dmitry Kiesewetter, Sergey Krivosheev, Sergey Magazinov, Victor Malyugin, Sergey Varzhel, Elizaveta Loseva, Sauletbek Koshkinbayev, Nurzhigit Smailov

**Affiliations:** 1 Higher School of High Voltage Engineering, Peter the Great Saint Petersburg Polytechnic University, 195251 St. Petersburg, Russia; 2 Research Institute of Light-Guided Photonics, ITMO University, 197101 St. Petersburg, Russia; 3 Department of Electronics, Telecommunications and Space Technologies, Satbayev University, Almaty 050013, Kazakhstan

**Keywords:** fiber Bragg grating, pulse elongation, spectrum, chirped Bragg grating, diagnostics, high-speed deformation

## Abstract

The pulsed elongation of fiber Bragg gratings is considered in order to be used to measure the displacement or deformation rate of objects. Optimal measurement modes were determined, numerical simulation of the output signal was performed during pulsed elongation or compression of the fiber grating, and the main patterns were analyzed. The results of the application of the Bragg gratings for the experimental determination of the deformation rate of materials under pulsed magnetic action are presented. Experimentally obtained and theoretical dependencies are compared. The dependencies of the change in the grating parameters—the coefficient and the half-width of the reflection spectrum with successive shortening of the grating—are given.

## 1. Introduction

Fiber Bragg gratings (FBGs) are widely used both in communication systems and as sensors of temperature, deformation of objects, pressure, refractive index of the environment, and other physical quantities. Therefore, a large number of scientific papers are devoted to the study of properties of FBG [1,2,3,4,5,6,7,8,9,10,11,12,13,14]. The basic principles of FBG operations are summarized, in particular in the reviews [1,2,3,4,13]; possible applications in [1,2,3,4,5,6,8,15,16,17]; and manufacturing technology in [1,2,3,7,11,13,14,15]. In particular, the use of FBG in medicine is considered in the reviews [4,6,8,11]; in biochemistry and pharmacy in [4,5,14]; for environmental applications in [5,11,14]; as well as for other applications [16,18,19,20,21,22,23,24,25,26]. An overview of works on the properties of chirped FBG and their application is given in [27].

It is known that the resonance wavelength $\lambda_{B}$ of the radiation reflected increases with the stretching of the FBG, which is due to a change in the period of the FBG, the refractive index and the diameter of the fiber core. For FBGs created in a standard quartz optical fiber (9/125 μm), a linear relationship takes place between the change in the wavelength $\Delta \lambda_{B}$ of the maximum spectral density of reflected radiation and the relative elongation of the FBG ε [1,7,28]:

(1) $$\Delta \lambda_{B}=k_{BE}\varepsilon$$

where $k_{BE}$ is the proportionality coefficient approximately equal to 1.2 · 10${}^{3}$ nm (12 nm per % or 1.2 · 10${}^{-3}$ nm per με) when the FBG is stretched at $\lambda_{B}$ 1550 nm and room temperature. It should be noted that $k_{BE}$ depends on the temperature of the FBG [29,30] and linearly depends on $\lambda_{B}$ [28].

The ability to measure the relative elongation by changing the value of $\Delta \lambda_{B}$ makes it possible to use FBGs as sensors of elongation and mechanical stress. However, all measurements of this type can be considered as measurements under stationary stretching or compression of FBGs: during the measurements, the parameters of the FBG do not change, and the FBG itself is elongated (or compressed) uniformly along its entire length, including for vibration and ultrasonic wave sensors [11,12,15]. With the pulsed stretching of an optical fiber with an FBG located at some distance from the impact site, the stretching of an FBG occurs with a delay and with a short pulse of stretching or compression; in addition, the structure of the FBG cannot be considered spatially homogeneous. The operation of sensors based on FBG under pulsed mechanical action has not been studied in sufficient detail.

Presumably, the only application for which pulsed mechanical action on FBG has been studied is the systems for shock wave and detonation diagnostics [17,22,24,31,32,33,34]. The basic concept [17] is that the change in the radiation reflection parameters (spectral and total power), which can be determined in real time during the physical destruction of FBG, occurs during the propagation of the detonation wave. The use of a chirped FBG gives a strong dependence of the spectral density of the reflected radiation on the length of the undestroyed part of the FBG, which simplifies the calculation of the required wave parameters. The use of high-speed interrogation for sensing in detonation and shock wave experiments is considered in [22,32]. In general, the determination of shock wave parameters can be performed without destroying the FBG by measuring changes in the spectrum of reflected radiation in real time [34,35,36]. These articles do not consider the elongation wave of an optical fiber with FBG passing a certain distance along the fiber before exposure to FBG, as well as simple physical and mathematical approximations and estimates for describing and numerically modeling the propagation of stretching or compression waves. This work is devoted to the study of such properties of FBG and their application for the research of pulsed mechanical action created by a pulsed magnetic field on electrical and structural materials.

## 2. Fundamental Principles

With a slow change in the FBG parameters, the change in wavelength is usually recorded using a source with a variable wavelength λ and a spectrally insensitive photodetector or using a source with a wide radiation spectrum and a spectrometer measuring the spectral density of reflected radiation (an interrogator). To register rapid changes in FBG parameters (occurring in microseconds and fractions of microseconds), the use of common interrogators cannot provide good accuracy in determining the dependence of the resonant wavelength on time, and it becomes necessary to use the power of radiation reflected by FBG at a fixed wavelength of the source as a measured value to determine the stretching of the optical fiber. The dynamic range of such measurements is significantly less than when using an interrogator, but the frequency band is significantly larger. The description of the fundamental principles presented below is also applicable for individual channels of multichannel systems and elements of the array of high-speed interrogator spectrometers. The measurement of pulse dependencies is most relevant for cases of mechanical action on the FBG, since the change in temperature in FBGs is a relatively slow process.

Let us consider the simplest model given below to study the basic patterns. Let the spectral power density of laser radiation ($p_{LD}$) and reflected FBG radiation ($p_{FBG}$) be described by Gaussian functions:

(2) $$p_{FBG}\left(\lambda \right)=A_{FBG}exp\left(-{\left(\frac{\lambda -\lambda_{FBG}}{\sigma_{FBG}}\right)}^{2}\right)$$

(3) $$p_{LD}\left(\lambda \right)=A_{LD}exp\left(-{\left(\frac{\lambda -\lambda_{LD}}{\sigma_{LD}}\right)}^{2}\right)$$

where $A_{FBG}$ and $A_{LD}$ are the normalization factors, $\lambda_{FBG}$ and $\lambda_{LD}$ are the central wavelength of the spectrum of reflection of FBG and laser radiation, and $\sigma_{FBG}$ and $\sigma_{LD}$ are the half-width of the spectrum of reflection of FBG and laser radiation. The following Gaussian approximation is used:

$$A_{FBG}=k_{r,FBG}P_{in},$$

$$A_{LD}=\frac{P_{0}}{\sqrt{\pi }\sigma_{LD}},$$

where $P_{0}$ is the laser radiation power in the optical fiber, $k_{r,FBG}$ is the maximum reflection coefficient of the FBG, and $P_{in}$ is the radiation power at the input of the FBG. Assuming the attenuation in the used segment of the fiber is negligible and the spectral sensitivity of the photodetector is constant within the range of the change λ, by performing integration along the wavelength, it is possible to obtain an analytical solution for the power value $P_{p}$ of the reflected FBG laser radiation, which will be recorded by the photodetector:

(4) $$P_{p}=\sqrt{\pi }k_{r,FBG}A_{LD}\frac{exp(-{\left(\lambda_{LD}-\lambda_{FBG}\right)}^{2}/\left({\sigma^{2}}_{LD}+{\sigma^{2}}_{FBG}\right))}{{\left(1/{\sigma^{2}}_{LD}+1/{\sigma^{2}}_{FBG}\right)}^{1/2}}$$

It is clear that the maximum power of the laser radiation reflected by the grating is achieved when the central wavelengths $\lambda_{0,FBG}$ and $\lambda_{0,LD}$ are equal. When increasing or decreasing $\lambda_{FBG}$, the value of $P_{p}$ decreases. If, in accordance with (1),

(5) $$\lambda_{FBG}=\lambda_{0,FBG}+k_{BE}\varepsilon$$

where $\lambda_{0,FBG}$ is the central wavelength of the spectral density of reflected radiation by FBG at zero elongation, then based on (4), it is possible to obtain the analytical dependence $P_{p}\left(\varepsilon \right)$. According to Formula (4), the dependence $P_{p}\left(\varepsilon \right)$ is also Gaussian. With the lengthening of the FBG (ε > 0), the nature of the change in $P_{p}\left(\varepsilon \right)$ depends on the position of the initial working point $\lambda_{0,FBG}$ relative to $\lambda_{LD}$. If the case is $\lambda_{0,FBG}<\lambda_{LD}$, then with the extension of the FBG, the power $P_{p}\left(\varepsilon \right)$ increases until the equality $\lambda_{FBG}=\lambda_{LD}$ is reached, and in the case of $\lambda_{0,FBG}>\lambda_{LD}$, it decreases. So, for example, with sinusoidal elongation, in the first case, the output signal (power $P_{p}$) will be in-phase with respect to the elongation, and in the second case, it will be in antiphase.

Let us introduce the notation:

(6) $$\sigma_{S}=\sqrt{{\sigma^{2}}_{LD}+{\sigma^{2}}_{FBG}}$$

Then, if we consider the small elongations at which $k_{BE}\varepsilon \ll \sigma_{S}$ occurs, we can assume that the dependence of $P_{p}$ is a linear function of ε, the proportionality coefficient of which depends on the position of the starting point (ε = 0). The minimum (theoretically zero) sensitivity to the change of ε takes place at the extremum point ($\lambda_{FBG}=\lambda_{LD}$). The theoretical dependence of the sensitivity to the change of ε, which can be defined as $\frac{{dP}_{p}}{d\varepsilon }$, has the form:

(7) $$\frac{{dP}_{p}}{d\varepsilon }=\frac{2exp\left(-\frac{{\left(k_{BE}\varepsilon +\lambda_{FBG}-\lambda_{LD}\right)}^{2}}{{\sigma^{2}}_{S}}\right)k_{BE}\sqrt{\pi }\left(k_{BE}\varepsilon +\lambda_{FBG}-\lambda_{LD}\right)}{{\sigma^{2}}_{S}{\left(\frac{1}{{\sigma^{2}}_{FBG}}+\frac{1}{{\sigma^{2}}_{LD}}\right)}^{1/2}}$$

The maximum sensitivity value is reached at the point

$$\Delta \lambda_{ms}=\pm \sqrt{({\sigma^{2}}_{LD}+{\sigma^{2}}_{FBG})/2},$$

(or $\pm \sigma_{S}/\sqrt{2}$), which can be obtained by equating to zero the second derivative of expression (4) taking into account (5):

(8) $$\frac{{d^{2}P}_{p}}{d\varepsilon^{2}}=\frac{2exp\left(-\frac{{\left(k_{BE}\varepsilon +\lambda_{FBG}-\lambda_{LD}\right)}^{2}}{{\sigma^{2}}_{S}}\right)k_{BE}\sqrt{\pi }}{{\sigma^{2}}_{S}{\left(\frac{1}{{\sigma^{2}}_{FBG}}+\frac{1}{{\sigma^{2}}_{LD}}\right)}^{1/2}}\left(-1+\frac{2{\left(k_{BE}\varepsilon +\lambda_{FBG}-\lambda_{LD}\right)}^{2}}{{\sigma^{2}}_{S}}\right)$$

and consider the limit for the case ε→0.

The value of $\Delta \lambda_{ms}$ is estimated to be 0.01% (or $10^{4}$με) for typical values of $\sigma_{FBG}$ and $\sigma_{LD}$ (for lasers with a DFB structure).

If the lengthening effect is applied to the input end of the optical fiber, in which the FBG is located at a distance of $L_{b}$ from the input end, then the change in the reflective properties of the FBG will occur with a delay of the amount indicated below:

(9) $$\Delta \tau_{SF}=L_{b}/\nu_{S}$$

where $\nu_{S}$ is the propagation velocity of the stretching wave in the fiber. The duration $\Delta \tau_{FBG}$ of the passage of the wave front through the FBG with a length of $L_{FBG}$ is $L_{FBG}/\nu_{S}$. If:

(10) $$\Delta \tau_{FBG}\ll \Delta \tau_{i}$$

where $\Delta \tau_{i}$ is the characteristic time of change of mechanical stress, then such an effect can be considered as quasi-stationary. In this case, the delay in changing the reflective properties of the grating can be calculated by the Formula (9), and the inhomogeneity of the FBG under this influence can be neglected.

If the values of $\Delta \tau_{FBG}$ and $\Delta \tau_{i}$ are approximately the same, and even more so if there is:

(11) $$\Delta \tau_{FBG}\gg \Delta \tau_{i}$$

then with such an effect, the FBG will no longer be uniform in length, i.e., for a short mechanical pulse, the FBG should be considered chirped. The change in the magnitude of the reflected radiation power with such a short exposure will be less than with a quasi-stationary one.

It should be noted that the propagation velocity of the acoustic wave $\nu_{s}$ in an optical fiber is significantly less than in a continuous medium. It can also be assumed that the acoustic wave propagates mainly through the protective coating of the fiber, and there is also a significant dispersion of the acoustic pulse during its propagation through the fiber. As will be shown below, the experimentally measured $\nu_{S}$ value is in the range from 2.5 to 3.8 km/s, depending on the tension of the optical fiber, the temperature of the fiber, the presence of bends, etc. The estimated value of $\Delta \tau_{FBG}$ is 3 μs at $L_{FBG}=10^{-2}$ m.

## 3. Measurement Methodology

The basis for numerical modeling and processing of the received signals are experimentally measured emission and reflection spectra of the semiconductor lasers and fiber Bragg gratings used. In the studies described below, DFB-type semiconductor lasers were used. The fiber Bragg gratings were inscribed using the optical scheme for the manufacture of a FBG based on a Talbot interferometer [37] and the Optosystems CL7500 KrF excimer laser system (manufactured in Troitsk), operating according to the master oscillator-power amplifier scheme [38,39]. Such system produces radiation with high spatial (>5 mm) and temporal coherence, which allows the use of a tunable Talbot interferometer to create a high-contrast interference pattern in the FBG inscription area. The SMF-28 standard optical fiber subjected to preliminary low-temperature hydrogen loading was used for the inscription of the FBG [40,41]. The use of the inscription technology described above makes it possible to manufacture high-performance Bragg reflectors of various types with a wavelength adjustment of the Bragg resonance [42].

The lengths of the manufactured fiber Bragg gratings were 10 and 15 mm. The parameters of the FBG were measured after the release of hydrogen from the optical fiber. The spectra were measured using an Anritsu brand spectrometer, model MS9740B. The obtained emission spectra of semiconductor lasers were approximated by the Gaussian function. The temperature of the semiconductor laser body was changed using the Peltier element and measured by the thermocouple. The following values of the parameters of the laser used in the work were obtained: the central wavelength of the radiation ($\lambda_{0,LD}$) at the operating temperature is 1050.6 nm, the half-width of the spectrum ($\sigma_{LD}$) is 0.0158 nm.

The spectral dependencies of the reflected radiation of FBGs were approximated by three functions:

(12) $$y=Aexp(-{\left(\frac{\lambda -\lambda_{0,FBG}}{\sigma_{1,FBG}}\right)}^{2})$$

(13) $$y=A{\left(\frac{sin(\left(\lambda -\lambda_{0,FBG}\right)/\sigma_{2,FBG})}{\left(\lambda -\lambda_{0,FBG}\right)/\sigma_{2,FBG}}\right)}^{2}$$

(14) $$y=A{\left(1-\frac{exp(2\left(\lambda -\lambda_{0,FBG}\right)/\sigma_{3,FBG})-1}{exp(2\left(\lambda -\lambda_{0,FBG}\right)/\sigma_{3,FBG})+1}\right)}^{2}=A\left(1-th^{2}\left(\frac{2(\lambda -\lambda_{0,FBG})}{\sigma_{3,FBG}}\right)\right)$$

The parameters of approximations of the spectral dependence of the FBGs—half-width and the standard deviation of the approximation from the measured dependence for the two FBGs—used in the work are shown in Table 1. From the data obtained, it follows that all the approximating functions give a relatively small standard deviation ($S_{a}$) of the determined parameters and can be used in further work. However, each of them has its own specific application, in particular, function 2 (13) allows you to take into account the presence of lateral maxima of the reflection spectrum of the FBG, if such exist, and function 1 (12) allows you to obtain analytical expressions when calculating the reflected radiation power. The use of more complex approximations, such as those considered in the review [7], is not required to solve this problem, since the integral value is of interest and not the spectral position of the peak.

## 4. Numerical Simulation of Pulsed Mechanical Action

The simplest simulation of the pulse stretching of the FBG was carried out in the approximation of uniform stretching. The time delay $\Delta \tau_{p}$ between the moment of the beginning of the pulse action at the input end of the optical fiber and the moment of the beginning of the stretching of the grating was calculated based on the propagation velocity of the deformation wave, and the stretching of the FBG was assumed to be spatially homogeneous along the entire length of the FBG from the moment of reaching (beginning) FBG stretching waves.

Let the relative elongation at the input end of the fiber created by the loading system under the action of a magnetic field on the sample under study be determined by the expression:

(15) $$E\left(\tau \right)={\left(sin\left(\tau_{a}\tau \right)exp\left(-\tau \tau_{a}/\tau_{d}\right)\right)}^{2}$$

where τ is the time, $\tau_{a}$ is the scale factor, and $\tau_{d}$ is the decay decrement. Expression (15) describes an effect that creates only a wave of fiber stretching and does not create a compression wave. Formally, the delay $\tau_{p}$ in modeling can be taken into account using the time offset $t=\tau +\tau_{p}$. Taking into account that (1):

(16) $$\lambda_{FBG}=\lambda_{0,FBG}+k_{BE}E\left(t\right)$$

(17) $$V\left(t\right)=\int_{-\infty }^{\infty }s_{LD}\left(\lambda \right)s_{FBD}\left(\lambda ,t\right)d\lambda$$

Using, for example, approximation (13):

(18) $$s_{LD}=A_{LD}{\left(\frac{sin(\lambda_{0,LD}-\lambda )}{\sigma_{2,LD}\left(\lambda_{0,LD}-\lambda \right)}\right)}^{2}$$

(19) $$s_{FBG}=A_{FBG}{\left(\frac{sin(\lambda_{0,FBG}-\lambda_{LD}+\alpha k_{BE}E\left(t\right))}{\sigma_{2,FBG}\left(\lambda_{0,FBG}-\lambda_{LD}+\alpha k_{BE}E\left(t\right)\right)}\right)}^{2}$$

by setting the values $\sigma_{2,LD},\sigma_{2,FBG},\lambda_{0,LD},\lambda_{0,FBG},\alpha$, where α is the normalization factor for expression (15) specifying the elongation, by performing integration (17) numerically, one can obtain the desired dependence V(t). For the Gaussian approximation of the laser spectra, taking into account Formulas (4) and (5), the expression for the output signal can be presented in analytical form:

(20) $$V\left(t\right)=\sqrt{\pi }\frac{exp(-{\left(\lambda_{LD}-\lambda_{0,FBG}-\alpha k_{BE}E\left(t\right)\right)}^{2}/\left({\sigma^{2}}_{LD}+{\sigma^{2}}_{FBG}\right))}{{\left(1/{\sigma^{2}}_{LD}+1/{\sigma^{2}}_{FBG}\right)}^{1/2}}$$

**Figure 1:**
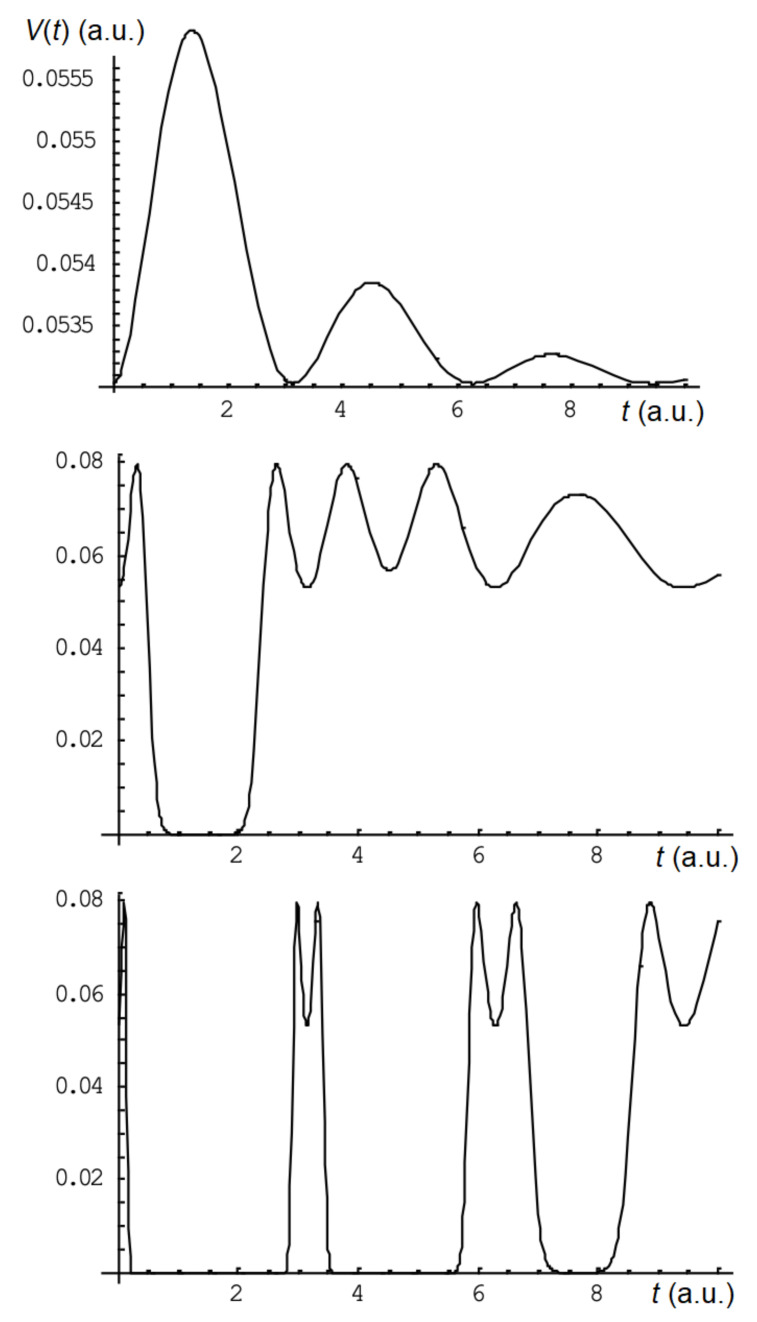
The output signal obtained by numerical simulation for small (α=10), medium (α=200), and large (α = 10,000) elongation (scales in arbitrary units).

Expression (20) also allows you to analyze all the basic patterns similarly to the numerical calculation given above. As an example, in the Figure 1 shows the calculated output waveforms at $\sigma_{LD}=0.15$ nm, $\sigma_{FBG}=$ 0.047 nm, $\lambda_{0,LD}=$ 1551.0 nm, $\lambda_{0,FBG}=$ 1550.9 nm, $\tau_{a}=$ 1 a.u., $\tau_{d}=$ 5 a.u. for conditionally small (α=10), medium (α=200) and large (α=10000) elongation. The specified parameters of the laser radiation spectrum and the reflection of the FBG are selected in order to obtain the most visual dependencies of the output signal.

The simulation results confirm the main patterns discussed (noted) above. At relatively small impacts, i.e., at a small value of the relative elongation ($\alpha k_{BE}E\left(t\right)\ll \sigma_{LD}$), the waveform at the output of the system repeats the dependence of the elongation on time, if the operating point (i.e., $\lambda_{0,FBG}$) does not coincide with the wavelength of the maximum spectral density of the laser ($\lambda_{0,LD}$). If there is $\lambda_{0,FBG}<\lambda_{0,LD}$, then the lengthening of the FBG leads to an increase in the signal at the output of the system. If there is $\lambda_{0,FBG}>\lambda_{0,LD}$, then the lengthening of the FBG leads to a decrease in the signal, i.e., the signal will be inverted relative to the time dependence of the elongation.

For the conditionally average elongation at the front of the elongation pulse (15), the signal initially increases until the condition $\lambda_{0,FBG}=\lambda_{0,LD}$ is reached and then decreases, possibly to zero, which corresponds to the condition $\lambda_{0,FBG}\gg \sigma_{S}$. When the elongation decreases (on the decline of the elongation pulse), the output signal occurs when the $\lambda_{FBG}$ returns to the sensitivity range $|\lambda_{FBG}-\lambda_{LD}|∼\sigma_{S}$.

The output signal differs from zero only at small values of the relative elongation at the front and the decay of the pulse at a conditionally large pulse elongation, i.e., when the dependence (15) has a value close to zero. The zero output signal corresponds to the condition $\lambda_{0,FBG}\gg \sigma_{S}$ similarly to the previous case.

It should be noted that the amplitude of the $V_{max}$ pulse signal for conditionally weak effects depends on the magnitude of the maximum elongation of $E_{max}$ (in Figure 1, item 1 is 0.02 units), and for medium and strong effects, the $V_{max}$ value is constant and does not depend on $E_{max}$ (in this case—0.8 units).

## 5. Measurement of the Rate of Tension Rise of an Optical Fiber with Bragg Grating

When using an FBG as a pulse elongation sensor for many practical applications, an important characteristic is the rate of increase in elongation $\xi_{el}$:

(21) $$\xi_{el}=\frac{d\varepsilon }{dt}$$

where *t* is the time. The value $\xi_{el}$ has dimension $s^{-1}$; however, it is more convenient to use the value με/s. If at the initial moment of time there is $\lambda_{LD}-\lambda_{FBG}>\left(2\ldots 3\right)\sigma_{S}$ and the change in the relative elongation $k_{BE}\varepsilon <3\sigma_{S}$, then with a uniform elongation of the fiber, the output signal will be an impulse of a close-to-Gaussian shape. The pulse duration at the level of $1/e(\tau_{1/e})$ corresponds to a change in the wavelength of the FBG by the value of the doubled half-width $\sigma_{S}$. Then, according to (1) and (21):

(22) $$\xi_{el}=2\sigma_{S}k_{BE}/\tau_{1/e}$$

If the value of ε during $\tau_{1/e}$ cannot be considered a constant value, then a more accurate estimate of $\xi_{el}$ can be obtained based on the half-width of the pulse duration $\tau_{1/e}$:

(23) $$\xi_{el}=k_{BE}\sigma_{S}/\tau_{h}$$

where $\tau_{h}=\tau max-\tau_{1/e}$ is the pulse duration between the points corresponding to the maximum of the signal and the signal level 1/e.

In some cases, the speed of movement of an object under pulsed mechanical action is of interest, which can be determined by the speed $\nu_{f}$ of movement of the end of the light guide attached to the object under study. At low $\nu_{f}$ speeds, its determination is not difficult:

(24) $$\nu_{f}=\varepsilon L_{OF}/\tau_{m}$$

where $L_{OF}$ is the length of the stretched (or elongated) optical fiber, and ε is the change in elongation during measurement $\tau_{m}$. The condition of applicability (24) is a uniform distribution of elongation over the entire length of the optical fiber, i.e., the possibility of using a quasi-stationary solution.

If the stretching or compression wave is localized on a limited length of the fiber, then condition (24) is not fulfilled. For such a mechanical effect, the grating should be considered spatially inhomogeneous (chirped). A strict solution or modeling of the light reflection of chirped FBG or FBG exposed to a shock wave [16,17] is a serious difficulty. For the simplest estimation of the magnitude of the radiation reflection from an inhomogeneously elongated (or compressed) FBG, it is possible to neglect the phase of reflected waves from individual strokes of waves and use the summation of the magnitude of reflected radiation from different regions of the FBG, within which the parameters of the FBG can be considered constant, and the resonant wavelengths are different. The problem of using this approach is, as a rule, the lack of information about the reflection coefficient and spectral characteristics of various areas of the FBG. The experimental study of such properties of FBG is given below.

Let us introduce $\lambda_{s,e}$ as the difference of the resonant wavelengths at the beginning ($\lambda_{0,sFBG}$) and the end ($\lambda_{0,eFBG}$) of the selected area of the FBG with length $L_{s,e}$. To be able to perform the above assessment, it is necessary that the total reflected radiation power can be assumed to be additive relative to the radiation power reflected from each of the selected sites, and the inequality is also fulfilled:

$$\lambda_{s,e}>\sigma_{FBG}$$

Then, the estimated value of the minimum rate of rise (or decline) of the relative elongation $\xi_{el}$ in inverse seconds (1/s) for the application of such a model is determined by the expression:

$$\xi_{el}>\frac{\sigma_{FBG}\nu_{f}}{k_{BE}L_{s,e}}$$

If the value of the half-width of the radiation spectrum $\sigma_{sFBG}$, reflected from the FBG section, does not depend on the length of this section ($L_{s,e}$), then this also simplifies the task of modeling signals under pulsed action on the grating.

## 6. Experimental Data

**Figure 2:**
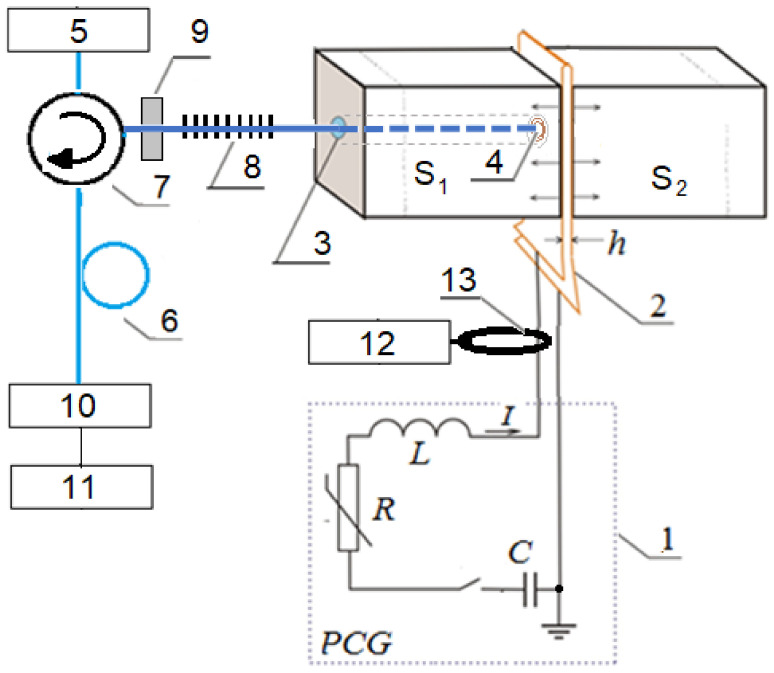
Scheme of the experimental setup: 1—the pulsed current generator; 2—conductor; $S_{1}$ and $S_{2}$—studied samples; 3—entrance hole; 4—the attachment point of the fiber with FBG to the sample $S_{2}$; 5—semiconductor laser with power supply unit; 6—optical fiber; 7—circulator; 8—fiber Bragg grating; 9—device that fixes the fiber; 10—photodetector module; 11—electronic unit; 12—oscilloscope; 13—Rogovski coil.

The electrical scheme for testing materials for uniaxial direct tension used in this work likes to the previously used one to study the mechanical properties of the TiNi alloy [43,44,45]. The experimental setup (Figure 2) consisted of pulsed current generator 1 (PCG), curved flat conductors 2, samples of the studied material $S_{1}$ and $S_{2}$, a fiber-optic system including the semiconductor laser 5 with power supply unit, photodetector module 10, circulator 7 with optical fibers 6 and fiber Bragg grating 8, as well as electronic unit 11 performing conversion (ADC), signal processing, and display of measurement results on the personal computer. The fiber containing the Bragg grating was located inside the hole of sample $S_{1}$ and conductors 2. The end of the fiber with FBG was attached to the inner surface of the test sample $S_{2}$ at point 4. The distance from the fixation point of fiber 4 to FBG was 0.5 m. The specified value of the initial stretching of the fiber was provided by the fixing device 9. The total length of fiber 8 was approximately 1 m. The surfaces of conductor 2 were separated by electrical insulation thickness *h*. The waveform of the current was recorded using the oscilloscope 12 and Rogovski coil 13. The parameters of the main PCG elements were as follows: the storage capacitor was 14.5 µF, the operating voltage was up to 30 kV, the intrinsic induction of PGG was 100 nH, and the wave resistance was 0.08 Ω. The shock load was formed under the action of a pulsed magnetic field of flat parallel copper conductors on which the pulse current generator is discharged. Parameters of current pulses: duration—1–5 microseconds; the maximum value of the current in the pulse—10–100 kA. The created pressure was transferred to the sample made of the special shape in such a way that its deformable part was subjected to uniaxial direct stretching [44,45].

The pressure pulse formed in the flat conductors was calculated from the obtained current waveforms. This pressure was transferred to the sample made in a special shape in such a way that the deformable part was subjected to uniaxial direct stretching [44,45]. An important advantage of the described method is the possibility of regulating the strength and duration of exposure to the material under study [46,47,48], and the disadvantage is the presence of strong electromagnetic interference, which makes it difficult to use common electronic sensors to measure the parameters of such exposure [49,50].

**Figure 3:**
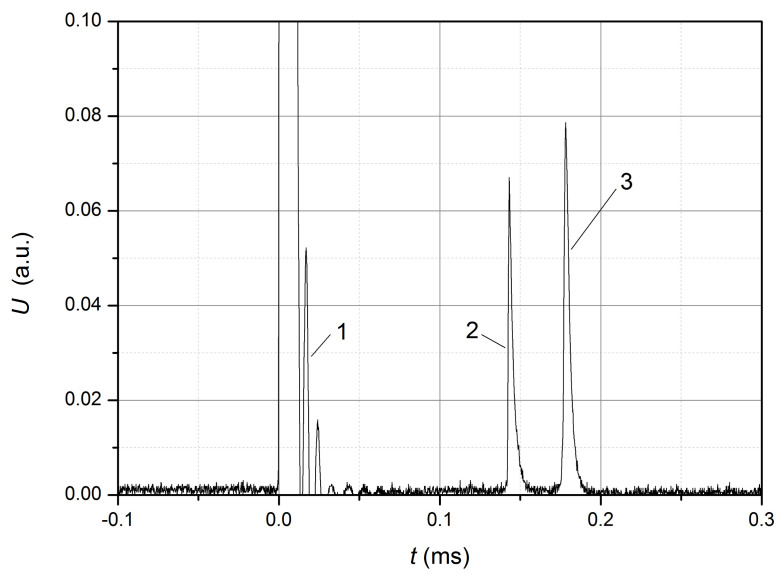
Waveform of the output signal: 1—induced interference caused by the current pulse; 2 and 3—pulses corresponding to the front and decay of the stretching wave.

**Figure 4:**
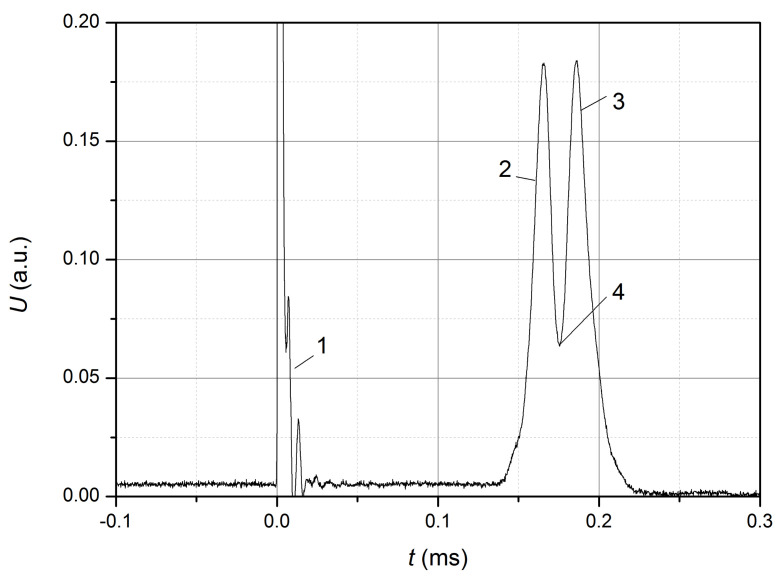
Waveform of the output signal: 1—induced interference caused by the current pulse; 2 and 3—pulses corresponding to the front and decay of the stretching wave; 4—the transition point from fiber stretching to compression.

Examples of the received waveforms are shown in Figure 3 and Figure 4.

Thus, the pulses shown in Figure 3 correspond to the induced interference (dependence 1) caused by the current pulse, from the front of which the time starts counting on the waveforms, as well as the pulses corresponding to the front and the decay of the stretching wave (dependence 2, 3) passing through the FBG. The specified waveform was obtained on the optical fiber having initially some tension, such that the operating point was near zero on the front of the Gaussian dependence U(ε) and an increase in ε led to an increase in the output signal *U*. For the given waveform, the delay between the front of the current pulse and pulse 2 (Figure 3) was 139 microseconds; between the maxima of pulses 2 and 3–35 microseconds, the duration of pulse 2 from level 1/e at the pulse front to the pulse maximum is approximately 0.4 μs. That is, the propagation velocity of the stretching wave along the optical fiber with a protective shell was approximately 3.6 km/s, and the estimated value of the rate of expansion of the stretching, assuming $\sigma_{S}=0.068$, was 200 με/s.

The waveform shown in Figure 4 was obtained in the absence of the initial tension of the optical fiber, and the fiber itself was partially bent. Since the pulse has two maxima, it can be concluded that the pulse front 2 and the decay 3 correspond to the front and decay of the stretching wave passing through the FBG, and point 4 corresponds to the transition from fiber stretching to compression, i.e., the rate of change ε at this point is 0. For this waveform, the delay between the front of the current pulse and pulse 2 (Figure 4) was 153 μs between the maxima of pulses 2 and 3–21 μs, and the pulse duration from the level 1/e at the front of the pulse 2 to the maximum of the pulse is approximately 5 μs, which allows us to assume a significant increase in the dispersion of the pulse. Since the time required to achieve a fiber stretching value equal to the tension in the previous case considered is not known for sure, the estimation of the wave propagation velocity based on $L_{b}$ and $\tau_{SF}$ can give a significant error. The estimated rate of increase in stretching at the pulse front is 16 με/s for this case and should be regarded as an equivalent value (close to the average value) due to non-fulfillment of the condition ν=const.

**Figure 5:**
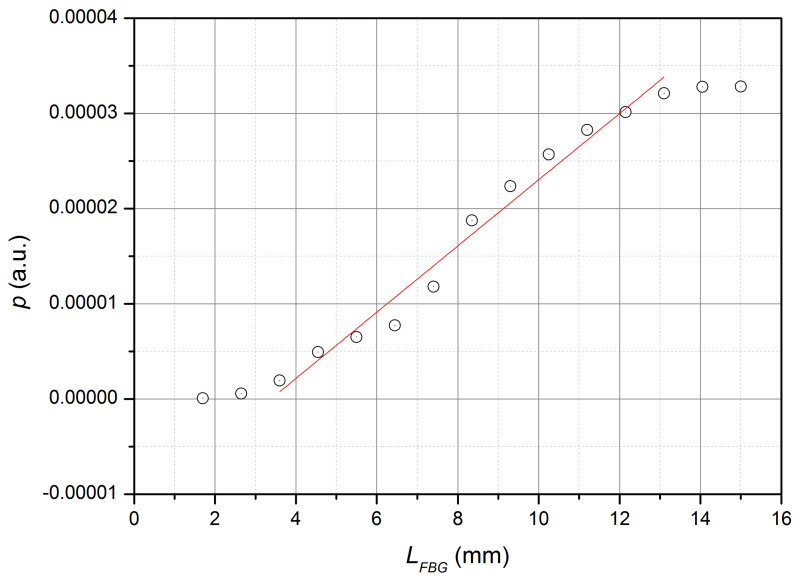
Dependence of the maximum spectral density of reflected radiation on the FBG length (experiment and linear approximation in the length range of 3–13 mm).

**Figure 6:**
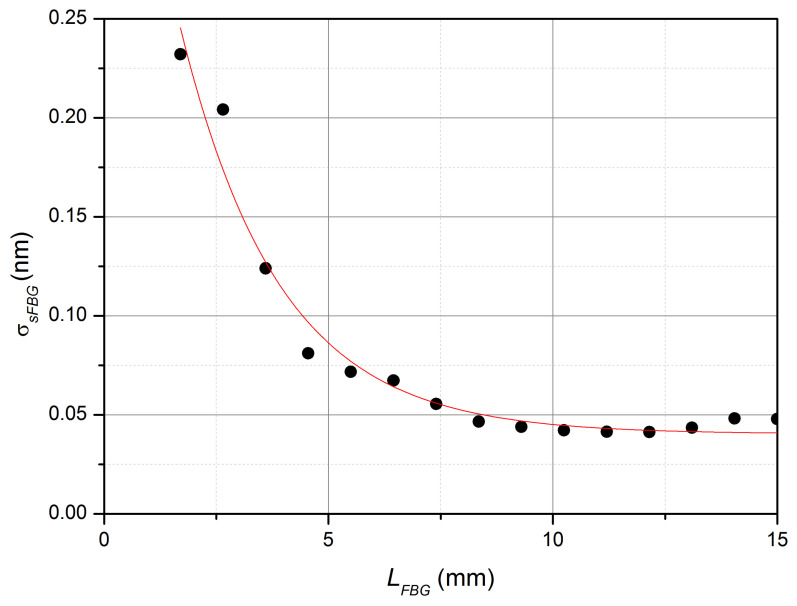
Dependence of the half-width of the reflected radiation spectrum on the FBG length (experiment and approximation by decaying exponential function with the constant component).

The experimental study of the spectral characteristics of the reflection on the limited area of FBG was also carried out. For this purpose, a 15 mm long FBG was made, and then, after the hydrogen was released from the FBG, the grating was successively shortened by the chipping method by approximately 1 mm and the reflection spectrum was measured at each length of the FBG. The obtained data—the value of the maximum spectral radiation density ($p(L_{FBG})$) and the half-width $\sigma_{sFBG}$ of the spectrum as function of the length of the FBG—are shown in Figure 5 and Figure 6.

It follows from the data obtained that the dependence $p(L_{FBG})$ is nonlinear, but the linear approximation of $p(L_{FBG})$ can be used as the simplest approximation in the range from 3 mm to 13 mm. That is, the condition of additivity of the reflected radiation power from the length of the FBG is approximately fulfilled in the specified range of lengths of the FBG. In addition, as the simplest approximation, the half-width of the reflection spectrum of the FBG can be assumed to be a constant when the length of the FBG is more than 7 mm. The resonant wavelength of the reflection of the FBG with the change in the length of the FBG from 2.5 mm to 15 mm changed by no more than 0.05 nm, which is presumably caused by a change in the distribution of internal mechanical stresses and the bending radius of the FBG during its shortening. It can be assumed that all gratings manufactured using the technology described above have approximately the same parameters.

## 7. Conclusions

Despite the fact that the use of FBG as a sensor of pulsed mechanical action, rigorously, is not non-invasive, such devices can be used to determine the parameters of pulsed mechanical action. An important advantage of using FBGs for these purposes is their insensitivity to pulsed electromagnetic fields, which makes it possible to use FBGs to determine the parameters of mechanical deformation of surfaces or objects under the action of pulsed electromagnetic action or in electromagnetic fields with high intensity. To determine the parameters for relatively small elongations or contractions, it is advisable to choose the operating point of the system in accordance with expressions (7) and (8) and for large ones at the initial section of the dependence V(ε).

It follows from the results obtained that the recorded rate of increase in elongation, determined by the parameters of the output signal pulse, can be commensurate with the speed of sound propagation in an optical fiber, and the parameters of the laser and the FBG used must be matched with the parameters of the measured process. In this paper, the general patterns of operation of the system based on a single FBG under pulsed tension or compression are considered. For more accurate measurements, more advanced systems should be developed and used, in particular using multiple FBGs.

## Figures and Tables

**Table 1:** Coefficients of approximation of FBG spectra.

	$L_{\mathrm{FBG}},$ mm	$\lambda_{0,\mathrm{FBG}},$ mm	$\sigma_{1,\mathrm{FBG}},$ nm	$S_{a}$	$\sigma_{2,\mathrm{FBG}},$ nm	$S_{a}$	$\sigma_{3,\mathrm{FBG}},$ nm	$S_{a}$
FBG 1	10	1550.59	0.066	$3\cdot 10^{-4}$	0.040	$3\cdot 10^{-4}$	0.060	$6\cdot 10^{-4}$
FBG 2	15	1550.85	0.068	$4\cdot 10^{-4}$	0.040	$4\cdot 10^{-4}$	0.061	$6\cdot 10^{-4}$

## Data Availability

Not applicable.
